# PML Nuclear Bodies and Cellular Senescence: A Comparative Study of Healthy and Premature Aging Syndrome Donors’ Cells

**DOI:** 10.3390/cells13242075

**Published:** 2024-12-16

**Authors:** Eugene Y. Smirnov, Sergey A. Silonov, Eva A. Shmidt, Aleksandra V. Nozdracheva, Nadezhda M. Pleskach, Mirya L. Kuranova, Anastasia A. Gavrilova, Anna E. Romanovich, Irina M. Kuznetsova, Konstantin K. Turoverov, Alexander V. Fonin

**Affiliations:** 1 Institute of Cytology of the Russian Academy of Sciences, Tikhoretsky av.4, 194064 St. Petersburg, Russia; e.smirnov@incras.ru (E.Y.S.); silonovsa@incras.ru (S.A.S.); evashmidt28@gmail.com (E.A.S.); shurupchi@mail.ru (A.V.N.); pleska@mail.ru (N.M.P.); miryakuranova@gmail.com (M.L.K.); asultanbekova@incras.ru (A.A.G.); imk@incras.ru (I.M.K.); kkt@incras.ru (K.K.T.); 2 Resource Center of Molecular and Cell Technologies, St-Petersburg State University Research Park, Universitetskaya Emb. 7-9, 199034 St. Petersburg, Russia; a.romanovich@spbu.ru

**Keywords:** aging, age-related diseases, senescence, cellular aging, PML nuclear bodies, LLPS, membrane-less organelles

## Abstract

Natural aging and age-related diseases involve the acceleration of replicative aging, or senescence. Multiple proteins are known to participate in these processes, including the promyelocytic leukemia (PML) protein, which serves as a core component of nuclear-membrane-less organelles known as PML nuclear bodies (PML-NBs). In this work, morphological changes in PML-NBs and alterations in PML protein localization at the transition of primary fibroblasts to a replicative senescent state were studied by immunofluorescence. The fibroblasts were obtained from both healthy donors and donors with premature aging syndromes (ataxia-telangiectasia and Cockayne syndrome). Our data showed an increase in both the size and the number of PML-NBs, along with nuclear enlargement in senescent cells, suggesting these changes could serve as potential cellular aging markers. Bioinformatic analysis demonstrated that 30% of the proteins in the PML interactome and ~45% of the proteins in the PML-NB predicted proteome are directly associated with senescence and aging processes. These proteins are hypothesized to participate in post-translational modifications and protein sequestration within PML-NBs, thereby influencing transcription factor regulation, DNA damage response, and negative regulation of apoptosis. The findings confirm the significant role of PML-NBs in cellular aging processes and open new avenues for investigating senescence mechanisms and age-associated diseases.

## 1. Introduction

Discovered by Hayflick more than 60 years ago [1], senescence is currently considered a major factor in the decline of tissue homeostasis and, consequently, other processes associated with organismal aging. Cellular senescence is a typically irreversible cell cycle arrest in response to telomere shortening and to various stress factors, including DNA damage and oxidative stress, leading to epigenetic remodeling [2]. During senescence, cells undergo extensive reorganization of signaling pathways, alterations in their transcriptome, disruption of genes related to cell cycle regulation and DNA replication/repair, modifications in the chromatin structure, and reorganization of cellular architecture [3,4,5,6]. Notably, senescent cells show activation of the p53–p21 and p16–RB signaling pathways, which leads to the suppression of genes necessary for proliferation, mitotic progression, chromatin integrity maintenance, and DNA damage repair and synthesis [6,7].

Senescence acceleration is considered as one of the main hallmarks of aging [8,9,10,11]. Also, senescent cells can accumulate in tissues, contributing to various age-related diseases, including cancer, fibrosis, and other pathologies associated with aging [12,13,14]. This process is mediated by the ability of senescent cells to produce and secrete various soluble molecules (factors) [15,16]. These secreted factors have been demonstrated to induce tissue inflammation or fibrosis and impair the functions of healthy neighboring cells, reducing the overall tissue regeneration capacity [17,18]. Due to its significant role in disease etiology, senescence is identified as a putative target for pharmaceutical intervention [19]. However, only a small number of studies have definitively demonstrated that senescent cells cause disease [20].

Among age-related diseases, there is a group of pathologies characterized by accelerated senescence and physiological aging (different progeroid syndromes, some neurodegenerative, and autosomal diseases). These diseases include Cockayne syndrome and ataxia-telangiectasia. DNA-repair-related progeroid Cockayne syndrome (CS) is a rare genetic disease characterized by signs of premature aging and progressive multi-organ dysfunction [21,22]. The molecular defect in CS occurs in the ERCC6 (CSB), ERCC8 (CSA), ERCC2, and ERCC3 genes, whose protein products participate in nucleotide excision repair [23,24]. Although the mechanisms linking DNA repair deficiency to CS symptoms are not yet clear, studies have shown that the CSB protein, a product of the ERCC6 gene, is also involved in DNA repair processes and transcription regulation in mitochondria [23,24].

Ataxia-telangiectasia (AT) is an autosomal recessive genetic disease characterized by genome instability, tissue degeneration, cancer predisposition, and premature aging. AT fibroblasts demonstrate signs of premature aging and high genome instability [25]. AT is caused by mutations in the ATM gene, which encodes the ATM kinase protein. ATM plays a crucial role in the cellular response to DNA damage, especially double-strand DNA breaks [25]. It has also been shown that in AT cells, the cGAS–STING pathway is involved in the induction of the senescence-associated secretory phenotype (SASP), which disrupts normal tissue function [26].

According to the current understanding, membrane-less organelles are biomolecular condensates formed via liquid–liquid phase separation (LLPS) that play a crucial role in the regulation and organization of intracellular processes [27]. Such organelles include PML nuclear bodies (PML-NBs) and multifunctional biocondensates involved in the regulation of various biological processes, including senescence [28,29,30].

Specifically, a PML-NB serves as a platform facilitating post-translational modifications of several proteins that play important roles in activating cellular senescence in response to telomere shortening and stress exposure, particularly p53 [28,31,32,33]. The acetylation of p53 protein within a PML-NB represents one of the key steps in activating p53-dependent senescence [34]. Various other senescence-associated protein post-translational modifications occur in a PML-NB, including ubiquitination and phosphorylation. It has been noted that PML participates in p53 activation through HIPK2-associated phosphorylation [35]. SUMOylation of client proteins recruited to stress granules plays a particular role in cellular senescence regulation [34,35,36,37,38]. Furthermore, studies have demonstrated that SUMO-2/3 overexpression can lead to premature cellular aging [39,40].

The scaffold protein of PML nuclear bodies, multifunctional promyelocytic leukemia protein (PML), is directly involved in replicative senescence processes. For instance, PML overexpression induces senescence in certain cell types [41]. PML also plays a crucial role in SASP activation and NF-κB regulation [42].

PML has at least seven main isoforms: one with nuclear-cytoplasmic localization (PML-I), five with nuclear localization (PML-II through PML-VI), and one with cytoplasmic localization (PML-VII) [43]. The functional diversity of PML protein extends beyond nuclear processes, as cytoplasmic PML isoforms demonstrate significant functional properties. For example, PML located in mitochondria-associated membranes (MAMs) participates in calcium transfer regulation between the endoplasmic reticulum and mitochondria, thus influencing apoptotic responses to various stimuli [44].

The direct involvement of both PML nuclear bodies and their scaffold protein PML in physiological and cellular senescence processes suggests their potential as promising markers for senescence, physiological aging, and accelerated aging. In this study, we correlated morphological changes in PML nuclear bodies and alterations in PML protein localization with widely used cellular senescence markers (telomere length, β-galactosidase activity) during the transition of primary fibroblasts to a senescent state. These fibroblasts were obtained from healthy donors and patients with accelerated aging syndromes (ataxia-telangiectasia, Cockayne syndrome).

## 2. Materials and Methods

### 2.1. Cell Lines Isolation and Cultivation

Primary dermal human fibroblast cell lines were isolated from skin biopsies obtained from the forearms of healthy young (3 years old) and older adult (75 years old) donors, as well as donors exhibiting symptoms of Cockayne syndrome (7 years old) and ataxia-telangiectasia (10 years old). Skin biopsy samples were obtained at the Pokrovsky Stem Cell Bank and the Pediatric Medical University in Saint Petersburg, Russia, and approved by the bioethical committee at the Institute of Cytology RAS (Protocol 13, 24 December 2021). All patients provided informed consent, and all procedures were conducted in accordance with relevant guidelines and regulations.

Cell lines were cultured in a humidified incubator at 37 °C with 5% CO_2_. The culture medium consisted of Eagle’s MEM (Biolot, St. Petersburg, Russia) supplemented with L-glutamine (Biolot, St. Petersburg, Russia), 10% fetal bovine serum (Capricorn, Düsseldorf, Germany), penicillin, and streptomycin (Biolot, St. Petersburg, Russia). The cell lines were passaged upon reaching 90% confluency. Each cell culture passage was accompanied by the cryopreservation of a cell portion in liquid nitrogen. Cultured cell lines were analyzed using β-galactosidase staining and measurement of telomere length when the cultures did not reach 90% confluency in 7 days.

### 2.2. β-Galactosidase Staining Protocol

The studied cell lines seeded on 35 mm Petri dishes (Jet Biofil, Guangzhou, China) were washed twice with PBS and then fixed in 3% paraformaldehyde for 3 min, followed by three PBS washes. The cells were then incubated for 16 h at 37 °C with a staining solution containing 40 mM citric acid/Na phosphate buffer (Sigma-Aldrich, St. Louis, MO, USA), 5 mM K_4_[Fe(CN)_6_]_3_H_2_O (Sigma-Aldrich), 5 mM K_3_[Fe(CN)_6_] (Sigma-Aldrich), 150 mM NaCl (Sigma-Aldrich), 2 mM MgCl2 (Sigma-Aldrich), and 1 mg/mL X-gal (neoFroxx, Einhausen, Germany). The cells were washed three times with distilled water, and a 70% glycerol solution was added for the evaluation of stained cells. Staining results were analyzed using a Carl Zeiss LSM 5 PASCAL fluorescence microscope by counting stained cells.

### 2.3. Immunofluorescence Staining

Cell lines were seeded onto glass slides to achieve approximately 60% confluency on the day of the staining. Cells were fixed with 3% paraformaldehyde (Sigma-Aldrich) for 10 min, followed by three washes with PBS. Cell permeabilization was performed using 0.1% Triton-X100 solution (Sigma-Aldrich) for 10 min, followed by three PBS washes. The cells were then incubated in blocking solution containing 3% BSA (Sigma-Aldrich) in PBS with 0.1% Tween 20 (Sigma-Aldrich) for 30 min. Following blocking, the cells were stained with anti-PML antibodies conjugated to AlexaFluor 647 (SantaCruz, sc-966, Dallas, TX, USA) for 2 h and washed three times with PBS containing 0.1% Tween. Subsequently, the cells were counterstained with DAPI (Sigma-Aldrich) and mounted in a mounting medium. Staining results were analyzed using confocal fluorescence microscopy.

### 2.4. Confocal Fluorescence Microscopy

Cell imaging following the immunofluorescent staining of PML protein was performed using an OLYMPUS FV3000 confocal microscope (60× oil immersion objective, NA 1.42). PML protein labeled with anti-PML antibodies conjugated to AlexaFluor 647 (SantaCruz, sc-966) was visualized by excitation with a 640 nm laser. Nuclear visualization was achieved using DAPI fluorescent dye with 405 nm laser excitation.

Image analysis of immunofluorescent staining was conducted using Fiji ImageJ software (version 1.54f) [45]. The StarDist plugin [46] was used for nuclear segmentation and size analysis. Subsequent detection and analysis of antibody-stained PML nuclear bodies, including quantification of bodies per cell, area measurements, and fluorescence intensity analysis, were performed using built-in ImageJ plugins.

### 2.5. Measurement of Relative Telomere Length

The relative telomere length was measured using quantitative polymerase chain reaction (qPCR) according to the protocol described by Joglekar et al. [47]. Genomic DNA was isolated using the ExtractDNA Blood & Cells kit (Evrogen, Moscow, Russia) following the manufacturer’s protocol. qPCR was performed using 5× qPCRmix-HS SYBR (Evrogen, Russia) with the following primers: telomere A-CGG TTT GTT TGG GTT TGG GTT TGG GTT TGG GTT and telomere B-GGC TTG CCT TAC CCT TAC CCT TAC CCT TAC CCT. The reactions were carried out using a BIORAD CFX96 Real-Time System. Data analysis was performed using BIORAD CFX Manager software (version 2.1).

### 2.6. Bioinformatic Analysis

Multiple databases were used for intersection analysis: the human PML protein interactome was obtained from the BIOGRID protein–protein interaction database v.4.4 (http://thebiogrid.org/, accessed on 1 August 2024); the PML-NB proteome database compiled from literature data has been previously described in our work [27]; CellAge and GenAge databases were used for senescence and aging data analysis, respectively [48,49]. Gene Ontology (GO) and KEGG pathway enrichment analyses were conducted using the Enrichr platform (https://maayanlab.cloud/Enrichr/, accessed on 1 August 2024), and the top 10 terms with the lowest *p*-values were selected [50]. Statistical significance was determined using Fisher’s exact test to evaluate nonrandom associations between categorical variables. FuzDrop [51], PSPredictor [52], and RIDAO [53] online predictors were used for LLPS analysis of the studied proteins according to our previously described work [27].

## 3. Results and Discussion

PML nuclear bodies (PML-NBs) are important participants in cellular responses to various types of stress and senescence. In this context, studying changes in the quantitative and qualitative characteristics of PML-NBs can enhance our understanding of senescence and aging processes.

### 3.1. Dermal Fibroblast Cell Line Establishment

**Figure 1:**
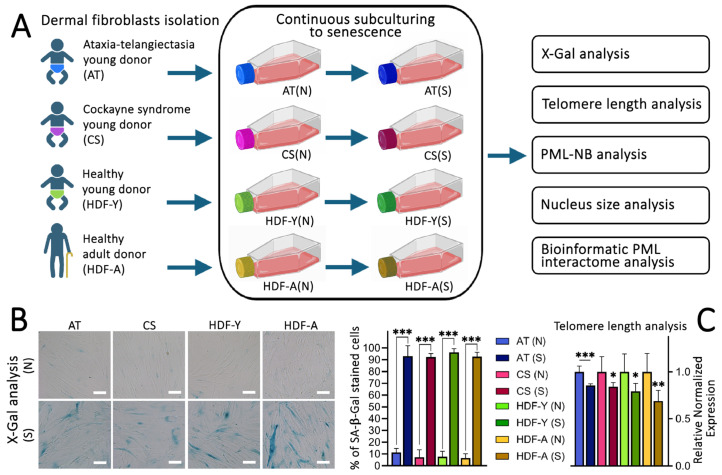
Establishment and characterization of primary fibroblast cell lines. (**A**) Experimental workflow showing the isolation of dermal fibroblasts from four donors: a young healthy donor (HDF-Y), an aged healthy donor (HDF-A), a young donor with ataxia-telangiectasia (AT), and a young donor with Cockayne syndrome (CS). Subsequently, the newly established normal cell lines (N) were subcultured until they reached replicative senescence (S) and were analyzed for multiple parameters. Schematics were created with BioRender.com. (**B**) Representative X-Gal staining images of cells from the young healthy donor and SA-β-Gal staining results for all cell lines. Scale bar: 50 µm. (**C**) Telomere length analysis by RT-PCR for all cell lines. Data represent the mean ± SD from ≥4 independent experiments. * *p* < 0.05, ** *p* < 0.01, *** *p* < 0.001.

In this work, four human dermal fibroblast (HDF) cell lines from different donors were used to investigate changes in the morphology and qualitative and quantitative characteristics of PML-NBs, as well as alterations in PML protein cellular localization. The HDF cell lines were obtained from donors who differed in age and diseases with premature ageing: a young healthy donor (HDF-Y), an aged healthy donor (HDF-A), a young donor with ataxia-telangiectasia (AT), and a young donor with Cockayne syndrome (CS). After early-passage cells were obtained, they were subcultured until they reached replicative senescence (Figure 1A). To confirm the achievement of replicative senescence, we used the classical histochemical method for detecting β-galactosidase activity using the X-Gal substrate at pH 6.0 (Figure 1B), known as ‘senescence-associated β-galactosidase (SA-β-Gal)’ [54]. Since telomere shortening is known to occur during senescence [55], the cells were additionally analyzed for telomere length changes using real-time PCR (Figure 1C). Both methods confirmed the achievement of replicative senescence. The obtained cells were used in subsequent comparative experiments.

### 3.2. Changes in PML-NB Characteristics

**Figure 2:**
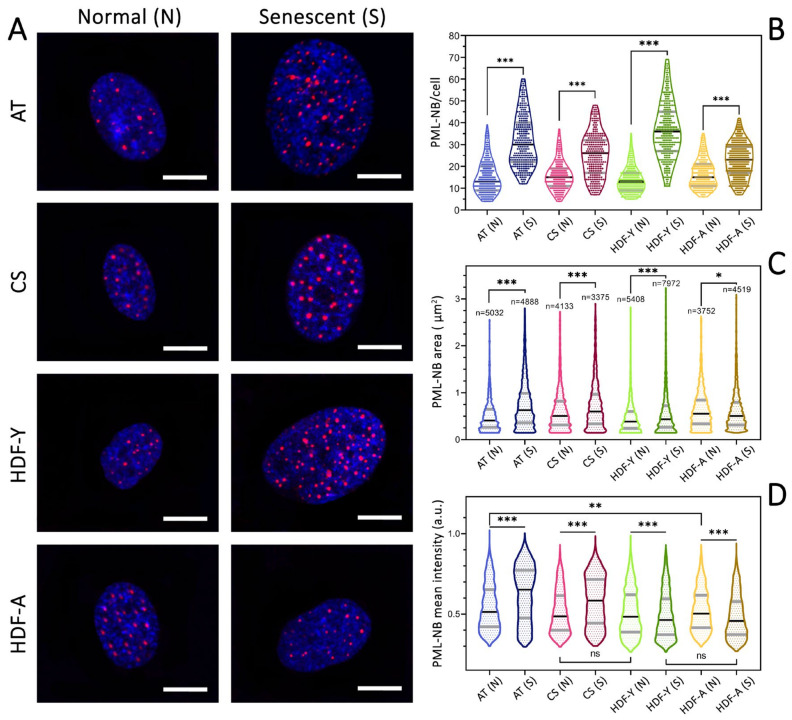
PML-NB characteristics in primary dermal fibroblast cell lines. (**A**) Representative confocal fluorescence microscopy images. Primary dermal fibroblasts from four donors: a young healthy donor (HDF-Y), an aged healthy donor (HDF-A), a young donor with ataxia-telangiectasia (AT), and a young donor with Cockayne syndrome (CS). Early-passage cells (N) and cells that reached senescence (S) are shown. Cells were stained with anti-PML antibodies conjugated with AlexaFluor 647 (red, excitation wavelength 640 nm) and nuclear stain DAPI (blue, excitation wavelength 405 nm). Scale bar: 10 μm. (**B**) Number of PML nuclear bodies (PML-NBs) per cell. (**C**) Area of individual PML nuclear bodies (PML-NBs) in the studied cell lines. (**D**) Mean intensity of individual antibody-stained PML-NBs. Results are shown as the mean ± SD from four independent experiments. * *p* < 0.05, ** *p* < 0.01, *** *p* < 0.001, ns—non significant.

Comparative analysis of the obtained cells using immunofluorescent PML staining revealed that the mean number of PML nuclear bodies (PML-NBs) per cell increases upon senescence across all studied cell lines (Figure 2A,B). The most pronounced difference in the mean PML-NB number per cell was observed in the fibroblast lines from the young healthy donor (HDF-Y). This finding suggests that the number of PML-NBs per cell could serve as a potential additional marker for cellular senescence. However, PML-NBs cannot be considered a definitive senescence marker, as PML-NB number increases may be associated with various stress conditions. For instance, a recent study [56] demonstrated a PML-NB number increase following irradiation.

The average PML-NB size increased in the senescence cells obtained from donors with accelerated aging disorders (Figure 2C). HDF-Y cell lines exhibited both a significantly smaller increase in PML-NB size upon senescence and an initially smaller PML-NB size compared to lines from other donors. The increase in both the number and the size of PML nuclear bodies may indicate a chronic stress state in fibroblast lines from the aged donor (HDF-A), the young donor with ataxia-telangiectasia (AT), and the young donor with Cockayne syndrome (CS), which aligns with the current understanding in this field [57,58,59].

Notably, the cell line from the aged donor (HDF-A) showed larger initial PML-NB sizes, and upon reaching senescence in comparison to other cell lines, it exhibited a slight decrease rather than an increase in average PML-NB sizes (Figure 2C). This phenomenon might be attributed to an increase in PML-NB numbers with insufficient initial PML protein levels during cellular transition to replicative senescence. This hypothesis also explains the decrease in the mean PML antibody fluorescence intensity during immunostaining of HDF-A lines transitioning to senescence (Figure 2D). It is worth noting that cells from the healthy donors also showed a slight decrease in fluorescence intensity (Figure 2D). This suggests that AT and CS cells exhibit more intensive PML protein accumulation within nuclear bodies upon reaching senescence compared to cell lines from healthy donors, resulting in increased fluorescence intensity. Additionally, AT and CS cells may undergo more intensive PML-NB fusion compared to cell lines from healthy donors, leading to a smaller increase in PML-NB numbers but a greater increase in individual PML-NB sizes. These findings confirm the significant role of PML-NBs in cellular aging and senescence processes and open new avenues for studying senescence mechanisms and age-associated diseases.

### 3.3. Nucleus Size and PML Protein Characteristics

**Figure 3:**
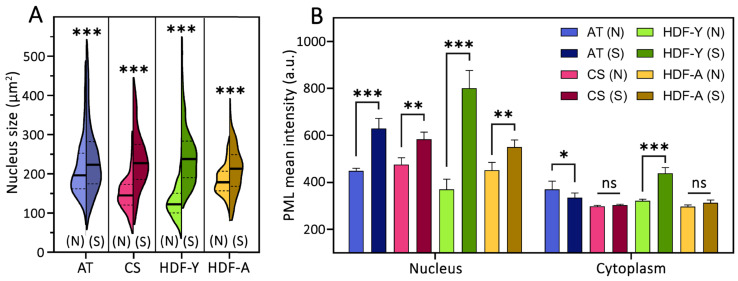
Nucleus size and relative immunofluorescence of antibody-stained PML protein in fibroblast cell lines. (**A**) Nucleus sizes of primary dermal fibroblasts from four donors: a young healthy donor (HDF-Y), an aged healthy donor (HDF-A), a young donor with ataxia-telangiectasia (AT), and a young donor with Cockayne syndrome (CS). Early-passage cells (N) and senescent cells (S) are shown. Data represent measurements of >1000 nuclei from three independent experiments. (**B**) Mean fluorescence intensity of antibody-stained PML protein in the nucleus and cytoplasm. The nucleus intensity is shown excluding PML-NB regions. Results are shown as the mean ± SD from four independent experiments. * *p* < 0.05, ** *p* < 0.01, *** *p* < 0.001, ns—not significant.

The nucleus size, which is currently recognized as one of the cellular senescence markers [19], increased in all studied cell lines during their transition to the senescent state (Figure 3A). Notably, the initial nucleus size of cells derived from the donor with ataxia-telangiectasia (AT) was larger than that of the nucleus of the other studied cell lines. The enlarged nucleus size in these dividing cells may be associated with the depletion or inhibition of ataxia-telangiectasia mutated protein (ATM), which leads to decreased lamin A expression and may consequently result in nuclear deformation [60]. Additionally, the nuclear size increase during senescence is hypothesized to be caused by cell flattening due to cytoskeletal element reconstruction [61]. ATM, in turn, can interact with F-actin and cytoskeletal factors [62].

The PML protein distribution in the nucleoplasm and cytoplasm was evaluated across the studied cell lines (Figure 3B). The mean PML fluorescence intensity (antibody-stained PML protein) in the nucleoplasm was found to increase upon senescence in all lines, with the most pronounced increase observed in HDF-Y. The mean PML fluorescence intensity in the cytoplasm increased during senescence only in HDF-Y. It can be hypothesized that in AT and CS, PML protein is more actively incorporated into PML-NBs, thus resulting in lower cytoplasmic levels, which correlates with the obtained data showing increased fluorescence intensity of individual PML-NBs (Figure 2D).

### 3.4. Bioinformatic Analysis

**Figure 4:**
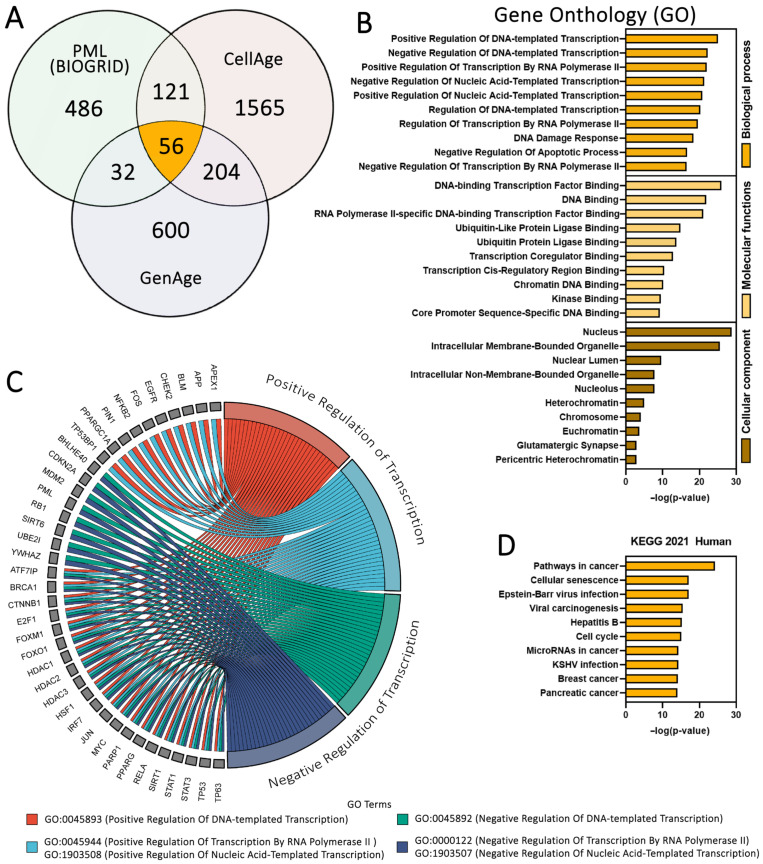
Analysis of the human PML protein interactome overlapping with senescence (CellAge) and aging (GenAge) protein databases. (**A**) Venn diagram showing the intersection between protein databases: the PML interactome from the BIOGRID database (light green), CellAge (light red), and GenAge (light blue). The orange segment represents 56 overlapping proteins identified across databases. (**B**) Gene Ontology (GO) enrichment analysis of 56 identified overlapping proteins. Categories shown include cellular components, molecular functions, and biological processes, ranked by enrichment score. (**C**) Chord plot representing the transcription factor activity of overlapping proteins. Analysis identified 39 of the 56 proteins as transcription factors. The chord plot was generated using SRplot [69]. (**D**) KEGG pathway enrichment analysis for 56 overlapping proteins. Pathways are ranked by −log10 (*p*-value).

The changes in the PML-NB and PML protein characteristics observed in this work suggest a relationship between senescence, aging, and molecular processes occurring within PML-NBs. To estimate the ratio of senescence- and aging-related proteins present in the PML protein interactome and PML-NB proteome, we performed a comparative analysis of several databases for overlaps. The BIOGRID database (v.4.4, http://thebiogrid.org/, accessed on 1 August 2024) was selected as the human PML protein interactome database; the PML-NB proteome database, described in our work [27], was compiled from literature data. CellAge and GenAge databases were selected for senescence and aging data, respectively [48,49]. Database analysis revealed that 30% of PML interactome proteins (209/695) and ~45% of PML-NB proteome proteins (92/205) are directly associated with senescence and aging processes (Figure 4A, Table S1). This result suggests extensive involvement of PML protein and PML-NBs in cellular and organismal aging processes.

For deeper investigation of the PML interactome proteins involved in cellular senescence and organismal aging (56 proteins overlapping across three databases; Figure 4A), we performed Gene Ontology (GO) analysis to identify their molecular functions, biological processes, and cellular components (Figure 4B). The analysis revealed that these proteins primarily function in transcription factor activation/inactivation, DNA damage response, and negative regulation of apoptosis. Notably, 39 of the 56 proteins are associated with positive or negative transcription regulation (Figure 4C), half of which show liquid–liquid phase separation (LLPS) propensity (Table S1). This suggests that the main function of PML-NBs in senescence and aging is transcription factor regulation through potential SUMOylation and other post-translational modifications. It is worth noting that many proteins from the 56 overlapping proteins are participants in the p53 signaling pathway, supporting a close relationship between PML, p53, and senescence [28,34].

KEGG pathway enrichment analysis of the 56 overlapping proteins (Figure 4D) showed significant involvement in three major functional clusters: cancer pathways, cellular senescence, and viral infections. Particularly noteworthy was the enrichment of pathways associated with three specific viruses: Epstein–Barr virus (EBV), hepatitis B virus (HBV), and Kaposi’s sarcoma–associated herpesvirus (KSHV). Each of these viruses demonstrates distinct interactions with PML-NBs and cellular senescence: EBV LMP1 protein suppresses cellular senescence [63], while Epstein–Barr capsid proteins colocalize with PML-NBs [64]; the HBV core protein undergoes SUMOylation and associates with PML-NBs, which is crucial for HBV rcDNA conversion [65]; HBV infection can induce hepatocyte senescence [66]; and KSHV can overcome replicative senescence in primary human lymphatic endothelial cells [67]. Significantly, during lytic infection, KSHV and EBV directly degrade PML via viral proteins, though there is no evidence that PML-NBs are disrupted by these viruses during latent infection [68]. Thus, PML-NBs may participate in more extensive molecular signaling pathways and play a more significant role in virus-controlled senescence than previously thought. The virus-specific patterns of PML-NB interaction suggest that these nuclear bodies may function as cellular sensors, integrating viral presence with senescence pathways.

## 4. Conclusions

This study demonstrates that cellular senescence is associated with changes in PML-NB counts across all investigated cell types. Moreover, the PML-NB size increases during transition to senescence in all cells except those from older adult donors, where proliferating cells already exhibit enlarged PML-NBs comparable to senescent cells. Significantly, cells from healthy donors show a slight decrease in the immunofluorescence intensity of PML antibodies localized in PML-NBs, while cells from diseased donors demonstrate increased immunofluorescence intensity. The most pronounced changes in the fluorescence intensity of PML antibodies localized in the nucleus and cytoplasm were observed at senescence in dermal fibroblasts from the young healthy donor. Thus, our data show differential changes in PML and PML-NB characteristics at proliferation and senescence across various cell types, with the PML-NB number per cell being the most significant characteristic at the senescent transition.

Bioinformatic analysis shows that 30% of PML interactome and 45% of PML-NB proteome proteins are involved in aging-related processes. Notably, transcriptional regulators represent the major functional group, half of which show liquid–liquid phase separation (LLPS) propensity according to bioinformatic predictions.

Taken together, these findings suggest that PML-NBs can be considered an additional marker of biological and cellular aging, participating in the regulation of these processes through the modulation of transcriptional activity.

## Data Availability

The original contributions presented in this study are included in the article/Supplementary Materials. Further inquiries can be directed to the corresponding author.

## Supplementary Materials

The following supporting information can be downloaded at https://www.mdpi.com/article/10.3390/cells13242075/s1, Figure S1. Western blot analysis for ATM protein. Anti-ATM antibody (Cell Signalling, cat. 2873S), Protein ladder (Thermo, cat. 26616); Figure S2. Ponseau S staining of the membrane; Table S1. Bioinformatic analysis.
