# Supplementary material for: PML Nuclear Bodies and Cellular Senescence: A Comparative Study of Healthy and Premature Aging Syndrome Donors’ Cells
Source: Cells. 2024 Dec 16;13(24):2075. doi: 10.3390/cells13242075 (PMC11674897; doi:10.3390/cells13242075)
Supplement: Supplementary file 1 [file cells-13-02075-s001.zip › Supplementary figure.pdf]

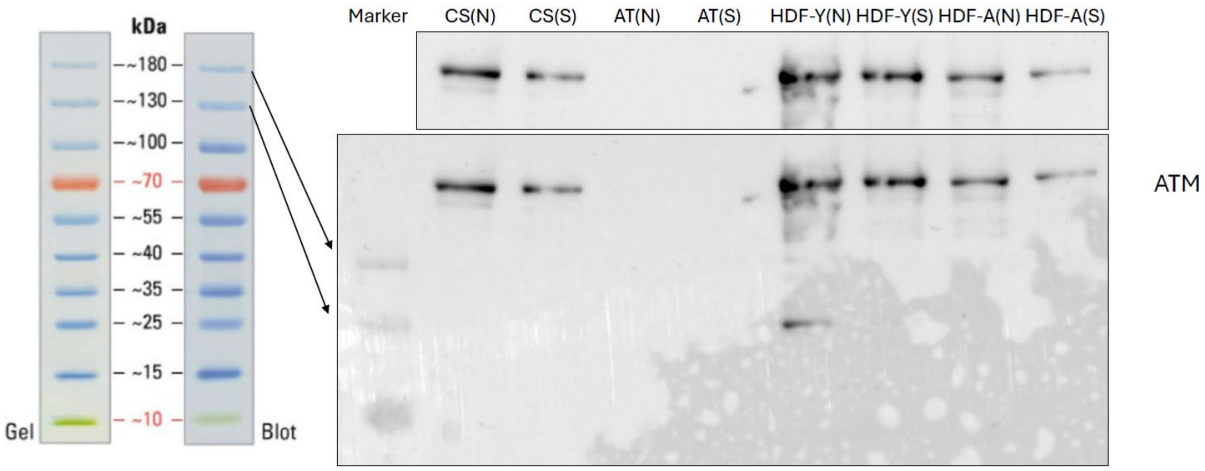

Figure S1 – Western blot analysis for ATM protein. Anti-ATM antibody (Cell Signalling, cat. 2873S), Protein ladder (Thermo, cat. 26616).

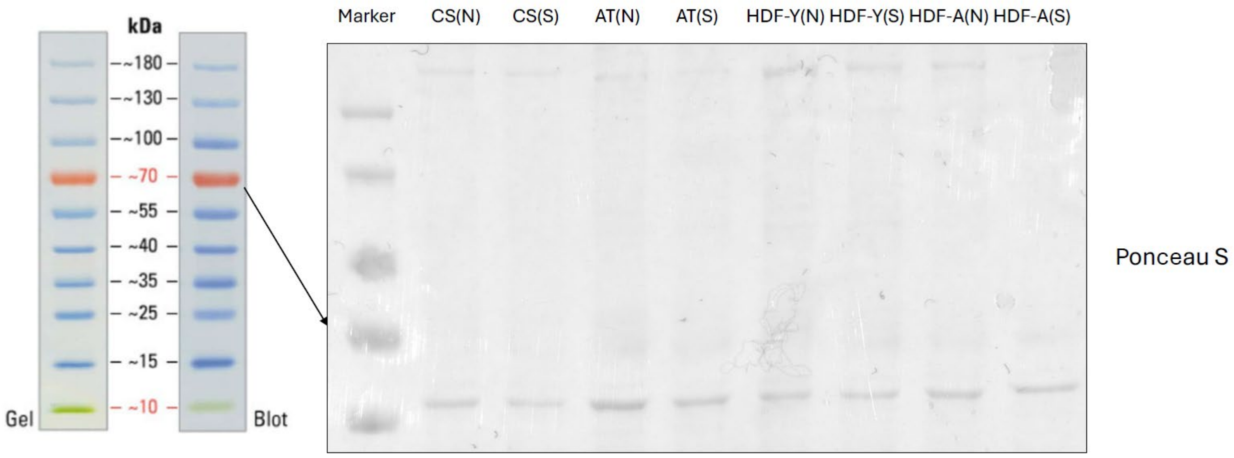

Figure S2 – Ponseau S staining of the membrane.
